# Formation Mechanisms and Phase Stability of Solid-State Grown CsPbI_3_ Perovskites

**DOI:** 10.3390/nano11071823

**Published:** 2021-07-14

**Authors:** Jessica Satta, Alberto Casu, Daniele Chiriu, Carlo Maria Carbonaro, Luigi Stagi, Pier Carlo Ricci

**Affiliations:** 1 Department of Physics, Campus of Monserrato, University of Cagliari, 09042 Monserrato, Italy; jessica.satta@dsf.unica.it (J.S.); daniele.chiriu@dsf.unica.it (D.C.); cm.carbonaro@dsf.unica.it (C.M.C.); 2 Nabla Lab, Biological and Environmental Sciences and Engineering (BESE) Division, King Abdullah University of Science and Technology (KAUST), Thuwal 23955-6900, Saudi Arabia; alberto.casu@kaust.edu.sa; 3 Laboratory of Materials Science and Nanotechnology, CR-INSTM, Department of Chemistry and Pharmacy, University of Sassari, Via Vienna 2, 07100 Sassari, Italy; lstagi@uniss.it

**Keywords:** CsPbI_3_, inorganic lead halide perovskites, solid-state synthesis, phase stability

## Abstract

CsPbI_3_ inorganic perovskite is synthesized by a solvent-free, solid-state reaction, and its structural and optical properties can be deeply investigated using a multi-technique approach. X-ray Diffraction (XRD) and Raman measurements, optical absorption, steady-time and time-resolved luminescence, as well as High-Resolution Transmission Electron Microscopy (HRTEM) imaging, were exploited to understand phase evolution as a function of synthesis time length. Nanoparticles with multiple, well-defined crystalline domains of different crystalline phases were observed, usually surrounded by a thin, amorphous/out-of-axis shell. By increasing the synthesis time length, in addition to the pure α phase, which was rapidly converted into the δ phase at room temperature, a secondary phase, Cs_4_PbI_6_, was observed, together with the 715 nm-emitting γ phase.

## 1. Introduction

Since the 1990s, organic–inorganic hybrid halide perovskites have received increasing attention in an optoelectronic context [1] for their intrinsic properties such as ambipolarity, high charge-carrier mobilities, great diffusion lengths and high absorption coefficients [2,3,4,5]. As a consequence, they found potential applications in many different sectors and devices from efficient light harvesters to photovoltaics, photodetectors and solar fuels [6,7,8,9]. In addition, due to their very high photoluminescent quantum yield and narrow-band emission in the whole visible region [10], they are promising materials for lighting and display applications.

Perovskites are a class of crystalline compounds described by the generic chemical formula AMX_3_, with A being a large cation, M a smaller metal cation and X an anion such as oxygen or one from the halide series. In hybrid halide perovskites, the organic cations are small and are typically restricted to methylammonium, ethylammonium and formamidinium; in all-inorganic halide perovskites, the A cation is caesium or rubidium. The metal cations are typically divalent metal ions such as Pb^2+^, Ge^2+^ and Sn^2+^, while the halide anions are I^−^, Cl^−^ and Br^−^. [11]

One of the most fascinating characteristics of perovskites is the possibility of tuning their optical and electronic properties by varying the composition of the constituent halide ions. On the other hand, their application in commercial devices is strongly hampered by low phase stability. The requirements of resistance to various environmental stresses such as humidity, illumination and high temperatures as well as being thermodynamically stable under operative conditions should be fully satisfied. Replacing the organic part of perovskites with inorganic materials currently represents a promising way of increasing long-term stability [12,13]. Indeed, the absence of organic components improves resistance to high temperatures and to the interaction with solvents and the surrounding atmosphere.

As a general rule, the structural stability of perovskites AMX_3_ is determined by the Goldschmidt tolerance factor and by the octahedral factor μ=rMrX, with *r_A_, r_M_, r_X_* being the different ionic radii. The ideal cubic structure is not very common and usually arises in the ranges 0.8 < *t* < 1 and 0.4 < *μ* < 0.9 ranges [14]. All of the members of the group of lead trihalides undergo phase transitions when varying the temperature, with the high temperature phase being the cubic perovskite. Indeed, for inorganic, lead-based CsPbX_3_ (X = Cl, Br, I), four phases are expected: a cubic phase (α), a tetragonal phase (β) and two orthorhombic phases (a black γ and a non-perovskite yellow phase). The α phase has a lower band gap as compared to the δ phase (in particular, 1.7 eV vs. 2.8 eV in CsPbI_3_, respectively) and displays a different luminescence emission peak (700 nm vs. 550 nm in CsPbI_3_) and lower emission efficiency. Because the spherical Cs^+^ ion is not large enough to preserve the PbI_6_ octahedra, CsPbI_3_ has a relatively smaller *t* value compared to the hybrid organic–inorganic perovskites [15]. Therefore, the cubic phase, which is desirable for optoelectronic applications, is stable only at high temperatures and undergoes a phase transition to the thermodynamically favored orthorhombic δ phase when the samples are cooled down to room temperature [15,16,17]:

$$t=\frac{r_{A}+r_{X}}{\sqrt{2\left(r_{M}+r_{X}\right)}}$$

Different synthesis techniques have been applied to obtain high-quality samples, with solution deposition techniques being the most widely utilized because of their low-cost and easy processing. There are mainly two approaches, either based on a one-step or a two-step procedure [18,19]. Mainly, dimethylformamide (DMF) and dimethyl sulfoxide (DMSO) are used as solvents, followed by a high-temperature annealing process. Solvent properties affect the quality of the film and, for this reason, antisolvent engineering or solvent evaporation control are used to promote the crystallization process [20,21]. Surface passivation strategies, the use of hydriodic acid or the so-called hydrogen lead iodide precursor are frequently employed to stabilize the cubic phase [22,23,24]. In a different approach, the cubic phase is achieved in solution by co-precipitation of monodisperse colloidal nanocrystals, with the stability being due to the large contribution of surface energy [25]. These methods require the use of long-chain organic capping agents and high boiling point solvents. It has been reported that the use of solvents and specific precursors can affect synthesis, creating organic inclusions in a fully inorganic perovskite [26]. Alternatively, other methods allow for producing lead halide perovskites without the exploitation of capping ligands or solvents, namely gas phase deposition, mechanochemical synthesis and synthesis of single crystals [27,28,29,30]. In this work, we focalized our studies on CsPbI_3_ obtained through a one-step, solid-state reaction. This kind of synthesis is relatively simple and, most importantly, does not need the use of any solvent or ligand. This point is fundamental for clearly assessing the structural parameters that define the stable phase and to determine how it is possible to achieve room-temperature, stable γ-phase CsPbI_3_. Recently, it has been proposed that many of the reported fully inorganic perovskites are stabilized by the organic part of the organic precursor utilized during the synthesis (i.e., hydrogen lead iodide), increasing uncertainty about the real structure of the samples [26].

On this basis, we investigated the formation of secondary phases by means of XRD, HRTEM, micro-luminescence and micro-Raman, imaging spectroscopy, time-resolved luminescence and optical measurements. We pointed out the key role of stoichiometry in defining phase formation.

## 2. Material and Methods

### 2.1. Materials

Caesium iodide (CsI, 50 ppm alkali metals, 99.9%) was purchased from Alfa Aesar (Ward Hill, MA, USA) and lead iodide (PbI_2_, 99%) was purchased from Sigma Aldrich (Saint Louis, MO, USA). All chemicals were used without any further purification.

### 2.2. Synthesis

CsPbI_3_ and Cs_4_PbI_6_ samples were synthesized through a solid-state reaction of CsI and PbI_2_. Stoichiometric raw materials were weighed, ground in an agate mortar and then sintered in a furnace.

$$\mathrm{CsI}+{\mathrm{PbI}}_{2}\to {\mathrm{CsPbI}}_{3}$$

$$4\mathrm{CsI}+{\mathrm{PbI}}_{2}\to {\mathrm{Cs}}_{4}{\mathrm{PbI}}_{6}$$

Different synthesis processes were performed.

First, the samples were slowly heated at 10 °C/min up to 400 °C and treated at a constant temperature for 10 min. Next, the samples were slowly cooled to room temperature.

The same process was carried out multiple times, treating the samples at 400 °C for 1 h, 5 h, 10 h, 17 h and 24 h, respectively, to study the effect of the synthesis duration on the final products. Hereinafter, the samples are called CPI3-10 m, CPI3-1 h, CPI3-5 h, CPI3-10 h, CPI3-17 h, CPI3-24 h. In order to discriminate the features of CsPbI_3_ from those of the secondary phases, we synthesized pure Cs_4_PbI_6_. We repeated the previous procedure, treating the sample at 400 °C for 5 h. The resulting sample will be called C4PI6 in the following work. As stated above, the stability was affected by oxygen and moisture, so the syntheses were performed in a continuous vacuum (~8∙× 10^−5^ mbar).

### 2.3. Characterization

X-ray patterns were collected at room temperature, using a Rigaku Miniflex II diffractometer with θ-2θ Bragg–Brentano geometry with Cu Kα (λ = 1.54059 Å) radiation. The powder patterns were recorded in the 5° ≤ 2θ ≤ 45° range. High temperature measurements were performed with a Bruker D8 Advance diffractometer operating at 30 kV and 20 mA equipped with a Cu tube (λ = 1.5418 Å), a Vantec-1 PSD detector (Freemont, CA, USA) and an HTK2000, Anton Paar (Graz, Austria) high-temperature furnace. The powder patterns were recorded in the 21° ≤ 2θ ≤ 45° range. To avoid the evaporation of the powder at high temperature, the sample was sealed in Kapton.

Micro-photoluminescence and micro-Raman imaging spectroscopy were collected using an MS750 spectrograph (sol-instruments, Mink Belarus) equipped with different gratings (150 gr/mm and 1200 gr/mm for luminescence and Raman measurements, respectively). The laser beam (405 nm and 785 nm for luminescence and Raman, respectively) was focalized through an Olympus objective (100×) with a laser power of about 0.75 mW. The measurements were acquired within a 100 ms time window in a 300–800 nm spectral range.

The micro-PL–Raman measurements were carried out with backscattering geometry coupled with a reflecting Bragg grating (Optigrate-Braggrade 405 and 785, respectively). Measurements were performed at room temperature with a spectral resolution for the Raman measurements of 1 cm^−1^.

Time-resolved photoluminescence (TR-PL) measurements were recorded by exciting the samples with 200 fs pulses sourced from an optical parametric amplifier (TOPAS-C, Light Conversion, Vilnius, Lithuania) pumped by a regenerative Ti:Sapphire amplifier (Coherent Libra-HE, Santa Clara, CA, USA). The repetition frequency was 1 kHz, and the TR-PL signal was recorded by a streak camera (Hamamatsu C10910) equipped with a grating spectrometer (Acton Spectra Pro SP-2300, Princeton Instruments, Trenton, NJ, USA). All the measurements were collected in the front-facing configuration to reduce inner filter effects. Proper emission filters were applied to remove the reflected contribution of the excitation light.

Excitation–emission fluorescence maps were recorded by a NanoLog spectrofluorometer (Horiba Jobin Yvon, Kyoto, Japan). Absolute quantum yields (QYs) were obtained by a quanta-ϕ-integrating sphere accessory with a 450 W Xenon lamp as the excitation source.

The absorption measurements were obtained by diffuse reflectance spectroscopy utilizing a UV-Vis-NIR Cary 5000, Agilent Technologies (Santa Clara, CA, USA). Measurements were performed using a PbS solid-state photodetector using KBr as reference. The reflection configuration measured the diffuse reflection of samples with respect to a reference sample, which was considered to have 100% reflectivity. The Kubelka–Munk equation was applied to define the absorption properties.

HR-TEM measurements were performed using a HRTEM JEOL 2010 UHR equipped with a Gatan imaging filter (GIF) with a 15-eV window and a 794 slow scan CCD camera. The structural characterization was conducted by 2-dimensional fast Fourier transform (2D-FFT) analysis, calculating planar and angular relationships between diffraction spots and comparing them with the diffraction cards previously adopted for X-ray diffraction analysis.

## 3. Results and Discussion

### 3.1. X-ray Diffraction Measurements

**Figure 1:**
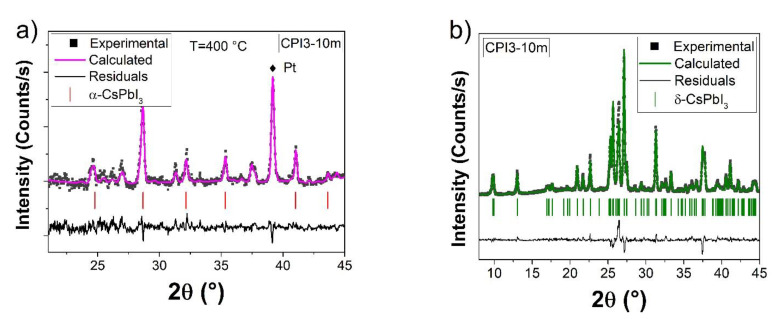
Rietveld refinement of sample CPI3-10 m at 400 °C (**a**) and at room temperature (**b**). ■ refers to Experimental pattern, color line is the pattern calculated by Rietveld refinement, in Black line the residuals between experimental and calculated pattern, the vertical bar indicates the theoretical peaks characteristic of α and δ-CsPbI_3_.

The phase stability of CPI3 samples at different temperatures is underlined in Figure 1. CPI3-10 m sample was obtained by solid state reaction with the correct stoichiometric amount of the precursors. Figure 1a reports the XRD pattern obtained at 400 °C in continuous vacuum, confirming the presence of α-CsPbI_3_, with lattice parameter a = 6.218(1) Å (ICSD 161481). The sample was kept at 400 °C degree for 10 min and cooled down to RT, hence the XRD pattern was collected again (Figure 1b). The sample turns in color from black to yellow and the Rietveld refinement on the X-ray diffraction pattern confirms the formation of the δ-CsPbI_3_ structure: the peaks are attributed to the orthorhombic δ-CsPbI_3_ (ICSD 27979), with crystal structure Pmnb (space group N. 62) and lattice parameters a = 4.7993(2) Å, b = 10.4521(6) Å, c = 17.7456(9) Å, with R_wp_ = 10.5% and R_B_ = 8%.

This result showed the possibility of rapidly synthesizing lead halide perovskites by solid-state reaction in the δ-CsPbI_3_ phase, which converted to α-phase only at a high temperature, as already reported in a previous study [31]. The presence of unreacted precursors could be excluded by the XRD analysis, which highlighted the higher amount of CsI (not PbI_2_) in samples with prolonged thermal treatment.

**Figure 2:**
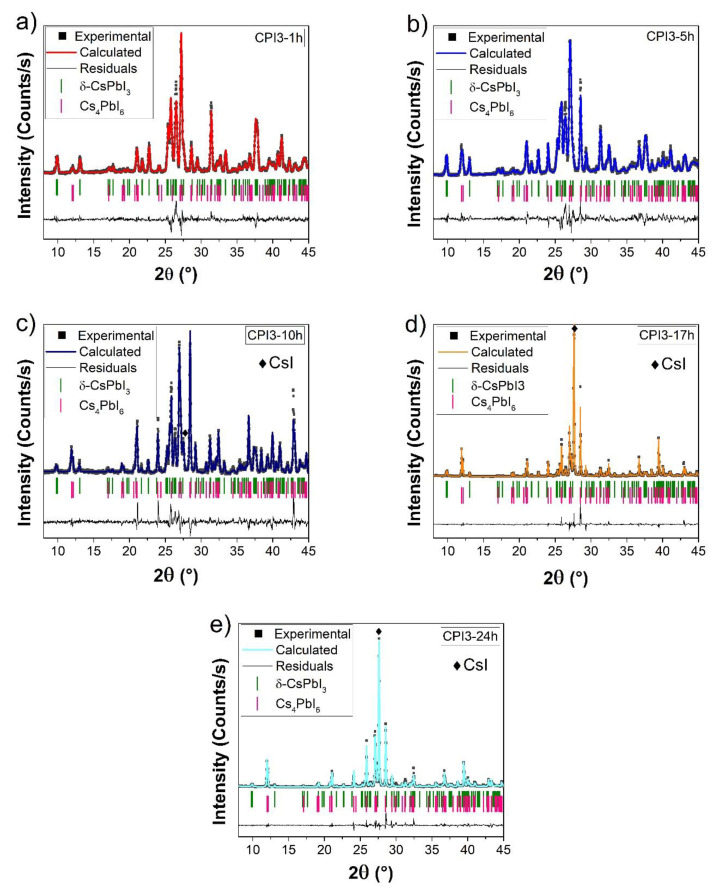
Rietveld refinement of CPI3 samples at different times: (**a**) 1 h, (**b**) 5 h, (**c**) 10 h, (**d**) 17 h, (**e**) 24 h. ■ refers to Experimental pattern, color line is the pattern calculated by Rietveld refinement, in Black line the residuals between experimental and calculated pattern, the vertical bar indicates the theoretical peaks characteristic of δ-CsPbI_3_ and Cs_4_PbI_6_.

The XRD patterns of samples obtained by prolonged synthesis at 400 °C (CPI3-1 h, CPI3-5 h, CPI3-10 h, CPI3-17 h, CPI3-24 h) were collected once the samples were brought back to RT (Figure 2). Comparing these patterns with the one related to CPI3-10 m, the secondary phase Cs_4_PbI_6_ with crystal structure R-3c:H (space group number 167) was clearly observable in addition to the δ-CsPbI_3_ phase. The percentages of these two phases were calculated with Rietveld refinement using the software MAUD (version 2.94, University of Trento, Italy) [32]. The analysis evidenced that the percentage of Cs_4_PbI_6_ increased proportionally to the synthesis duration (Table 1)

**Figure 3:**
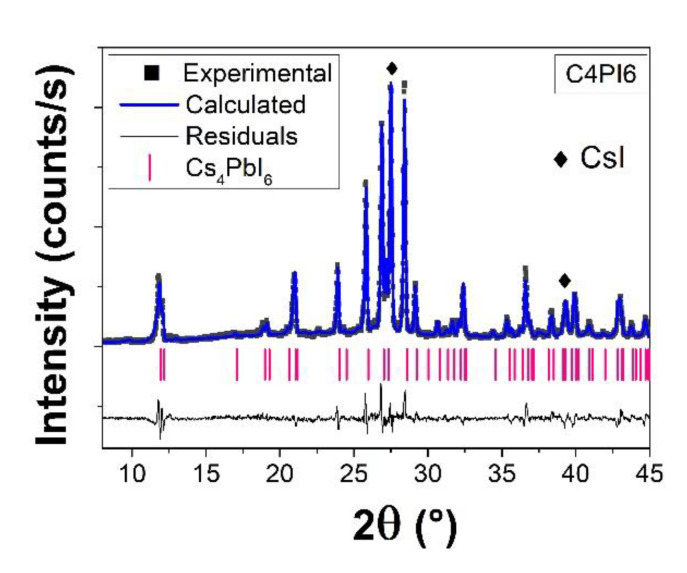
Rietveld refinement of C4PI6 sample. ■ refers to Experimental pattern, color line is the pattern calculated by Rietveld refinement, in Black line the residuals between experimental and calculated pattern, the vertical bar indicates the theoretical peaks characteristic of Cs_4_PbI_6_.

Additionally, Figure 3 reports the Rietveld refinement of the C4PI6 sample obtained from the synthesis of 4CsI + PbI_2_. The pattern revealed the presence of Cs_4_PbI_6_ up to 74% by weight, with a residual contribution of CsI precursor (20%) and 6% by weight δ-CsPbI_3_. The lattice parameters of Cs_4_PbI_6_ were a = 14.609(1) Å and c = 18.36(5) Å with R factors R_wp_ = 12.19% and R_B_ = 9.32%.

### 3.2. Luminescence and Raman Maps

To better understand the relation between the length of the synthesis and the presence of the secondary phase (Cs_4_PbI_6_), luminescence properties and Raman features were analyzed.

**Figure 4:**
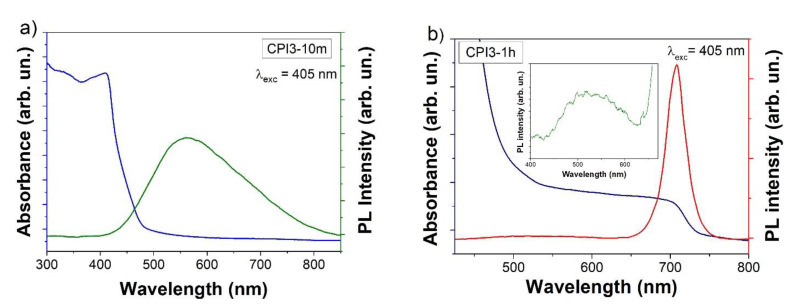
Absorption and steady-state luminescence spectra of the sample CPI3-10m, respectively in blue and green (**a**) and of the sample CPI3-1h, respectively in blue and red (**b**). The inset in figure (**b**) shows a zoom of the range 400–670 nm.

The sample obtained after 10 min showed the typical PL of the δ phase, a broad and weak emission around 550 nm (Figure 4a). The absence of CsI in the XRD measurement of sample CPI3-10 m strongly suggests that the emission from unreacted CsI doped with Pb can be excluded. (Figure 1). Increasing the synthesis duration, the luminescence started to become not uniform, and a strong emission appeared at around 715 nm, typical of black phases α and γ [25,33,34] (Figure 4b).

The broad emission from the δ-phase could be well reproduced with two contributions, while the red emission was the effect of a single band from the excitonic recombination in the α and γ phases (see Figure S1 and Table S1 in Supplementary Materials).

Similar features were observed in the samples obtained by prolonged synthesis (see Figure S2 and Table S2 in Supplementary Materials).

The local properties were, therefore, analyzed by optical microscope imaging coupled with a Raman and luminescence system. The samples appeared to be constituted of black spots surrounded by a white phase, mixed to a yellow phase.

**Figure 5:**
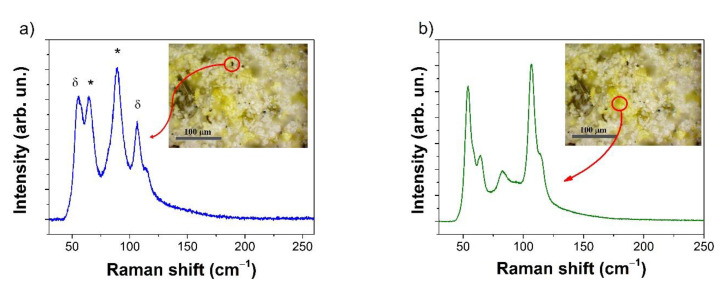
Raman spectra of CPI3-10 h sample, λ_exc_ = 785 nm. (**a**) Spectrum collected on a black spot, pointed out in the inset, with peaks in the Cs_4_PbI_6_ (*) and CsPbI_3_ δ phases. (**b**) Spectrum gathered on the yellow part of the sample, pointed out in the inset. The images were obtained with optical microscope imaging.

The Raman spectrum collected on the black spots showed the presence of Cs_4_PbI_6_ (65 and 90 cm^−1^) and δ-CsPbI_3_ (55, 107 cm^−1^). The yellow parts of the samples appeared to be δ-CsPbI_3_ (Figure 5) [31].

**Figure 6:**
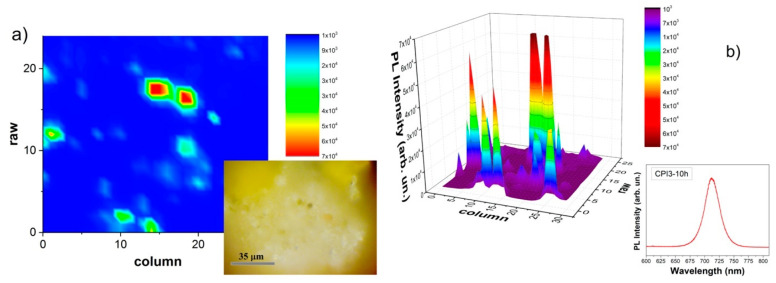
Luminescence map of the emissions at 715 nm of the sample CPI3-10 h: in (**a**), a 2D map and, in the inset, the image through optical microscope and in (**b**), a 3D map and, in the inset, the PL emission spectrum.

Focusing our attention on the Photo Luminescence at 715 nm, we performed a luminescence map. Figure 6. correlates the PL map and the optical imaging. It is possible to notice how the emission is more intense with correspondence to the black spots.

**Figure 7:**
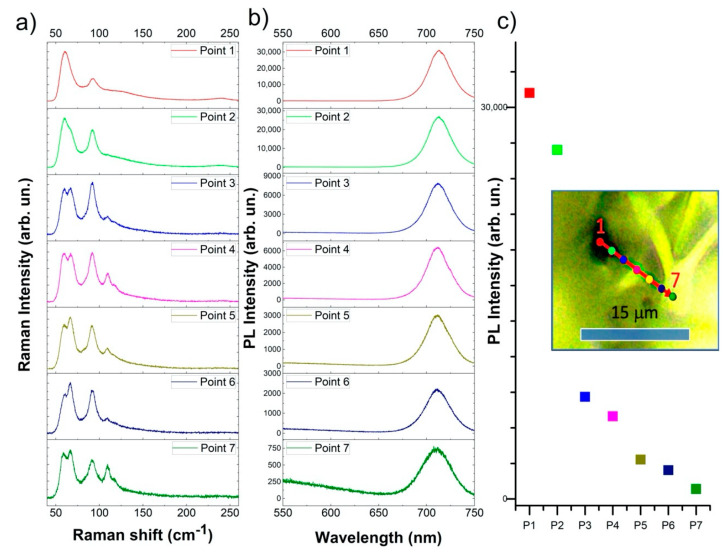
Raman and luminescence measurements on seven points in the CPI3-10 h sample. (**a**) Raman spectra, (**b**) luminescence spectra and (**c**) 715 nm PL intensity. The spectra were collected at a spatial point indicated in the inset of panel (**c**). The colour relates the Raman and luminescence spectrum, the intensity of the peak at 715 nm and the point in the map.

To better and deeper correlate the luminescence to a specific phase, we performed a series of measurements on 7 points (inset in Figure 7) close to an optically black point by simultaneously collecting Raman and luminescence spectra at each point. The black spot at point 1 showed an intense luminescence at 715 nm with a Raman spectrum typical of the γ-CsPbI_3_ and peaks at 60 and 240 cm^−1^ [30,35]. Moving from the black spot to the yellow region, the luminescence at 715 nm decreased in intensity (Figure 7b,c). Simultaneously, in the Raman spectra (Figure 7a), it was possible to observe the appearance of the secondary phase, Cs_4_PbI_6_ (65 and 90 cm^−1^), and finally, δ-CsPbI_3_ (58, 107 and 115 cm^−1^) with its related broad luminescence band at 550 nm.

The quantum efficiency of the samples calculated at 715 nm that were obtained with an integration sphere with a de-focalized beam gave a general overview of the emission properties of the different samples (Table 2). In addition, considering that this red emission was related to a γ-phase, and it was well known that the quantum efficiency of this phase was close to unity [36], it was possible to obtain an indirect rough estimation of the amount of γ-phase in the synthetized samples.

The values in Table 2 confirm the low amount of γ-CsPbI_3_ deduced by X-ray diffraction measurements below the 1% detection limit [36,37].

**Figure 8:**
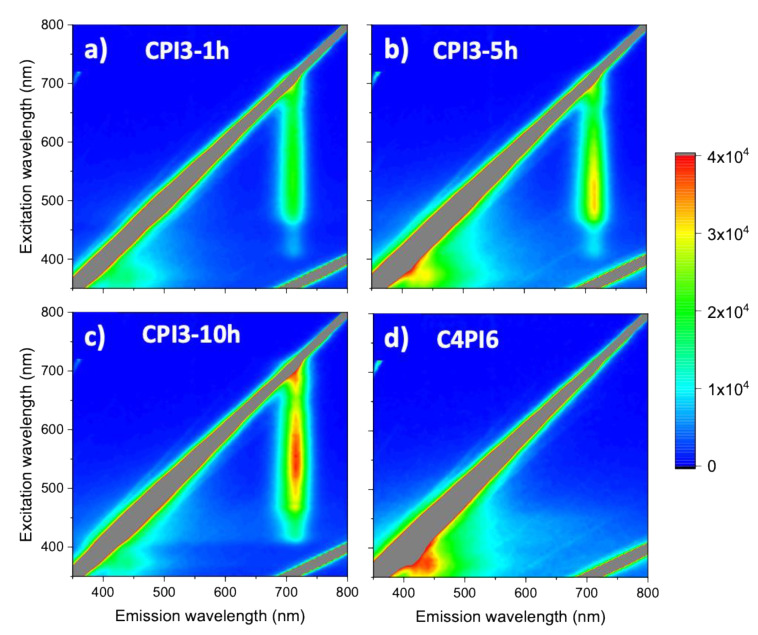
3D-Photoluminescence excitation spectra of different samples: (**a**) CPI3-1 h, (**b**) CPI3-5 h, (**c**) CPI3-10 h and (**d**) C4PI6.

In Figure 8, the photoluminescence excitation/emission maps of the samples treated at different synthesis time are reported. In Figure 8a–c, three CPI3 samples are shown, while Figure 8d displays, for comparison, the fluorescence map of the C4PI6 sample. Two different regions in the CPI3 samples could be clearly identified in the ultraviolet (UV) range (300–400 nm excitation, broad band 400–450 nm emission) and in the visible range (450–650 nm excitation, 715 emission) (see Figure S3 for an extrapolation of the excitation spectra of the emission at 715 nm). While the visible region was easily assignable to the γ-phase, the UV region could be assigned to the presence of the Cs_4_PbI_6_ phase by comparison with the map of the C4PI6 sample. However, due to the presence of CsI revealed by XRD measurements (see Figure 2 and Figure 3), whose emission properties lay in the same spectral range, a small amount of CsI:Pb could not be totally excluded [38]. Furthermore, the optical features of the δ-phase were not easily detectable, being overlapped by the broad emission of the Cs_4_PbI_6_ phase.

Time-resolved photo-luminescence measurements performed on the emission at 715 nm gave more insight into the properties of the recombination channel and consequently, on the intrinsic properties of the luminescent phase.

**Figure 9:**
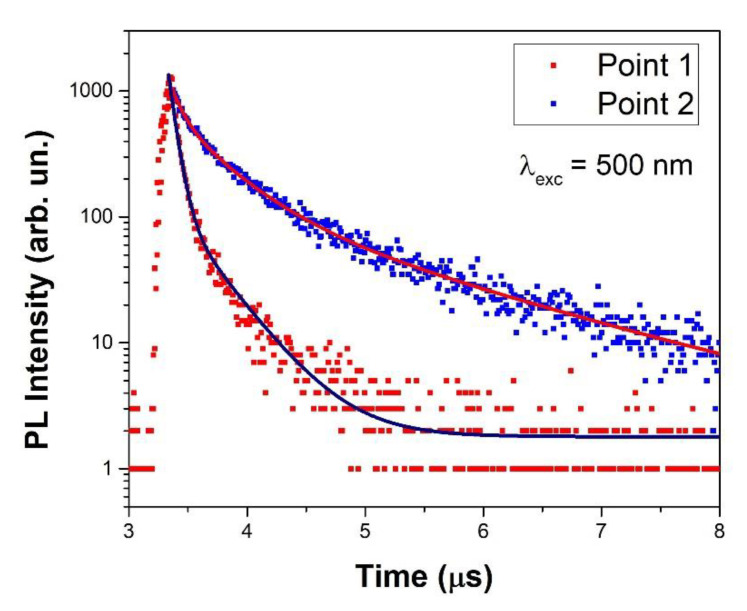
Time-resolved luminescence measurement at two different points of the CPI3-10 h sample; λ_exc_ = 500 nm.

Figure 9 shows the results of the measurement at two different points on the sample synthesized in 10 h. The sample was not uniform, and different lifetimes for the same emission at 715 nm were observed. The curves were fitted with a multi-exponential decay function:

I(t)=I0+∑i=1nAie−(t−t0)τi

with *I*(*t*) time-dependent PL intensity, *I*_0_ initial PL intensity, *A_i_* amplitude, *t* time, *t*_0_ initial time, τ_i_ the characteristic lifetime and *n* = 2 and *n* = 3 for points 1 and 2, respectively. Table 3 retrieves the fitting parameters that are in accord with previous results on CsPbI_3_ perovskites [22,39]. The average lifetime has been calculated using the following relation [40]:

$$\bar{\tau }=\frac{\sum_{i}A_{i}\cdot \tau_{i}^{2}}{\sum_{i}A_{i}\cdot \tau_{i}}.\;$$

Faster decay times from the same emitting centers were related to the presence of a non-radiative path:

(1) $$\frac{1}{\tau_{\mathrm{obs}}}=\frac{1}{\tau_{R}}+\frac{1}{\tau_{\mathrm{NR}}}=\gamma_{R}+\gamma_{\mathrm{NR}}$$

where τ_obs_ is the decay time experimentally measured by time-resolved luminescence analysis and τ_R_ and γ_R_ (τ_NR_ and γ_NR_) are the decay time and transition rate for a radiative (non-radiative) process. The faster the observed decay, the higher the presence of the non-radiative path often related to structural defects.

In this case, it was possible to argue that a defective matrix non-homogeneously generated a non-radiative path for the emissions from the γ−phase of CsPbI_3_, as could be evinced from the punctual Raman/luminescence analysis and from HRTEM images.

### 3.3. HRTEM and Epitaxy Studies

**Figure 10:**
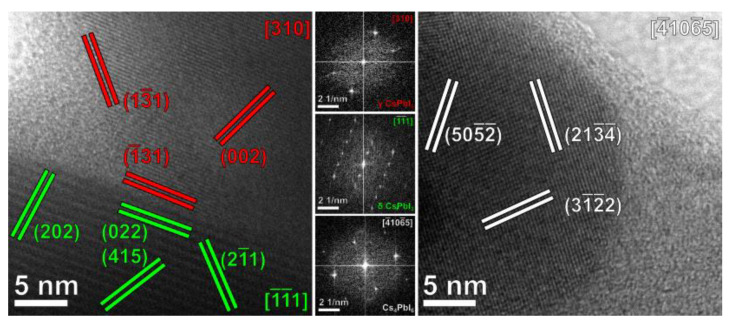
Representative HRTEM images of the CPI3-10 h sample. Lattice planes of γ-CsPbI_3_, δ-CsPbI_3_ and Cs_4_PbI_6_ are indicated in red, green and white, respectively. The 2D-FFT diffractograms used to calculate the orientations of each domain are reported in the central column.

High-resolution TEM (HRTEM) structural analysis provided a direct confirmation of the features of the CPI3-10 h sample, namely the presence of nanoparticles with multiple, well-defined crystalline domains of different crystalline phases and usually surrounded by a thin, amorphous/out-of-axis shell. Two dimensional Fast Fourier Transform (2D-FFTs) diffractograms were calculated for different regions of interest (ROIs), and the corresponding planar and angular relationships between diffraction spots were analyzed against diffraction cards of the phases previously identified by XRD analysis to understand the orientation of each domain. The results were compatible with the formation of crystalline domains of γ-CsPbI_3_, δ-CsPbI_3_ and Cs_4_PbI_6_, as reported in Figure 10, with lattice planes of each phase being indicated by different false colors.

Moreover, the well-defined straight interface between the γ− and δ-phases of CsPbI_3_, reported in Figure 10, was analyzed to verify the possibility of epitaxial symmetry relationships between the two phases. An interface alignment and a vector alignment were identified as follows:

δ-phase (0 2 2)//γ-phase (−1 3 1)

δ-phase [−1 −1 1]//γ-phase [3 1 0]

These symmetry relationships were used to calculate the actual lattice mismatch (*m*) occurring between average lattice spacings of the two phases along a given direction (Table 4). In general terms, the mismatch parameter *m* between the average lattice spacings of two different phases (indicated as d_1_ and d_2_, respectively) can be defined as:

m = 2 × ∣d_1_ − d_2_∣/(d_1_ + d_2_).

**Figure 11:**
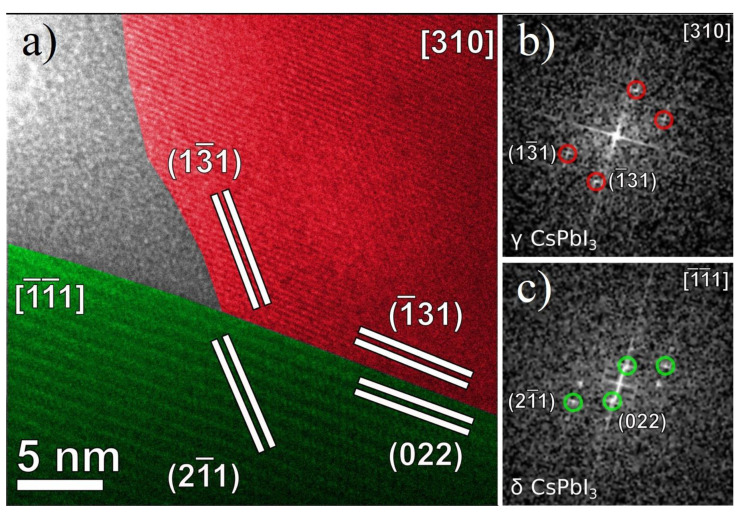
(**a**) Epitaxy study on the interface between γ-CsPbI3 and δ-CsPbI3, previously reported in Figure 10. γ-CsPbI3 and δ-CsPbI3 crystal domains in the HRTEM image are depicted in red and green, respectively. (**b**,**c**) The diffraction spots in the 2D-FFT diffractograms corresponding to the lattice planes used for the mismatch calculations are indicated according to the same color coding.

The Fourier analysis of the HRTEM images at the interface between two well-defined crystal domains of γ− and δ-phases was performed by 2D-fast Fourier transform (FFT) of equally-sized ROIs and proved their alignment as epitaxial, related domains: six unit cells of δ-CsPbI_3_ (6*) along the (0 2 2) direction corresponded to eight cells of γ−CsPbI_3_ (8*) along the (−1 3 1) direction, one unit cell of δ-CsPbI_3_ along the (2 −1 1) direction corresponded to one cell of γ−CsPbI_3_ along the (1 −3 1) direction and three unit cells of δ-CsPbI_3_ (3*) along the (−1−1 1) direction corresponded to 4 cells of γ−CsPbI_3_ (4*) along the (3 1 0) direction (see Table 4). The relevant epitaxially related lattice planes are presented in Figure 11 along with the corresponding diffraction spots in the 2D-FFTs.

A slight angular distortion could be observed between the (2 −1 1) spots of δ-CsPbI_3_ and the (1 −3 1) spots of γ−CsPbI_3_ in comparison to their theoretical value, which could be attributed to local deformations in the crystalline frameworks in proximity to the interface.

**Figure 12:**
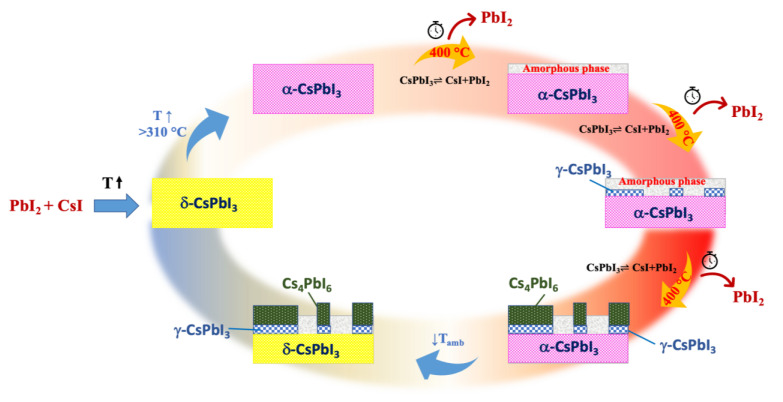
Model of the phase transition during the synthesis process and image of the sample after synthesis.

A general model for phase formation in solid-state synthesis is proposed in Figure 12. In the first step, δ-CsPbI_3_ is obtained from the precursors, CsI and PbI_2_, for temperatures below 300 °C. Next, as the temperature increases, the transition between the δ and α phase occurs in a relatively fast period (observed in 10 min).

The α-phase is a cubic CsPbI_3_ perovskite structure consisting of I ions corner-shared by two [PbI_6_]^4^ octahedra, with the Cs cation occupying the 12-fold coordination site formed in the middle of the cube of eight octahedra. The α-phase requires the correct stoichiometry in the perovskite structure and a high temperature (above 320 °C) to remain stable.

As time goes on, at 400 °C, the CsPbI_3_ phase partially undergoes a dynamic equilibrium consisting of its dissociation into the starting precursors, CsI and PbI_2_.

$$\mathrm{CsI}+{\mathrm{PbI}}_{2}\;⇌{\mathrm{CsPbI}}_{3}$$

PbI_2_ partially evaporates, leaving behind most/all of the CsI and creating an amorphous layer around α-CsPbI_3_. As already reported in the literature, an excess of CsI in the CsPbI_3_ helps the formation and the stabilization of the distorted perovskite γ phase [41]. The γ phase is orthorhombic, belonging to the Pbnm space group, and it remains stable at RT in a non-stoichiometric relationship CsI/PbI_2_ > 1. On the other hand, the α phase converts to the δ phase at RT [14,15].

The presence of CsI was evidenced in the XRD diffraction data reported and discussed in Figure 2. However, the higher percentage of CsI (not PbI_2_) was observed in samples with prolonged thermal treatment. This data confirmed the hypothesis that there was a dynamic equilibrium between the perovskite phase and CsI + PbI_2_.

Upon returning to RT after a variable amount of time spent at 400 °C, local variations in the CsI/PbI_2_ ratio, caused by the evaporation rates of PbI_2_, determine which stable phases will form in the samples. α phase crystals will form in regions with an optimal CsI/PbI_2_ ratio (i.e., =1) at a high temperature, and they will turn into δ phase at RT. Whenever the ratio is higher than 1, the creation of γ-CsPbI_3_ is favored over α. Finally, when the CsI/PbI_2_ ratio >>1, the formation of crystalline domains of secondary-phase Cs_4_PbI_6_ is most likely because the temporal increase of the thermal treatment generates a stronger excess of CsI, which prevents the formation of the perovskite phase and facilitates the formation of Cs_4_PbI_6_ (CsI/PbI_2_ about 4:1).

In this sense, structural analysis performed by HRTEM showed that domains of the different phases could be observed in close proximity, which confirmed the heterogeneity in sample composition previously revealed by optical microscopy, luminescence and Raman maps. In addition, the presence of epitaxial relationships at the interface between γ and δ phase domains suggested that the structural α → δ rearranging, caused by local variations in the PbI_2_ ratio, did not imply a drastic rearrangement of the atomic positions, so that a smooth structural transition between γ and δ phases could be maintained even with a sharp interface.

## 4. Conclusions

The paper is focalized on the solid-state synthesis of pure, inorganic lead halide perovskites of the class CsPbI_3_.

We showed that a pure α phase could be obtained with a brief treatment (in the order of minutes) at 400 °C without any solvent, and that it quickly passed to a stable δ phase at room temperature.

XRD analysis evidenced that an increase of treatment time at steady temperature generated the formation of the secondary phase Cs_4_PbI_6_, while micro-Raman and micro-luminescence measurements underlined the presence of the luminescent γ-phase.

The analysis of the collected data, time resolved measurements and HRTEM imaging permitted us to understand the phase behavior during the synthesis as a function of the temporal duration. After a few minutes at a high temperature, the CsPbI_3_ phase partially underwent a dynamic equilibrium process consisting of dissociation into its starting precursors, CsI and PbI_2_. Lead iodide quickly evaporated at 400 °C, creating an excess of CsI and the consequent formation of a stable perovskite γ-phase at room temperature and, in a longer temporal step, the secondary phase Cs_4_PbI_6_.

The samples were not uniform and presented amorphous phases and defectivity in the crystals’ structures but remained stable at room temperature.

Beyond the importance of base studies on this important class of materials, in this work, we have shown the possibility of easily obtaining bulk CsPbI_3_, and we indicate a mechanism for future development and control of the phase formation of lead halide perovskites.

## Figures and Tables

**Table 1:** Results of Rietveld refinement on CPI3 samples at room temperature.

Time	Phases		R_wp_	R_B_
	δ-CsPbI_3_	Cs_4_PbI_6_		
10 min	100%		10.5%	8.0%
1 h	90.6%	9.4%	12.1%	9.6%
5 h	54.9%	45.1%	12.6%	9.8%
10 h	42.1%	57.9%	13.6%	10.6%
17 h	33.2%	66.8%	12.8%	8.9%
24 h	21.5%	78.5%	17.6%	12.6%

**Table 2:** Quantum yield vs. synthesis time.

Sample	CPI3-1 h	CPI3-5 h	CPI3-10 h	CPI3-17 h	CPI3-24 h
Synthesis time	1 h	5 h	10 h	17 h	24 h
Quantum yield	0.6%	0.7%	1.86%	0.5%	0%

**Table 3:** Fit parameters of TR-photoluminescence measurements.

	$\bar{\tau }(\mu s)$	A_1_	τ_1_ (μs)	A_2_	τ_2_ (μs)	A_3_	τ_3_ (μs)
Point 1	0.144	1,823.671	0.060	131.900	0.346	-	-
Point 2	0.908	448.511	0.093	497.428	0.399	136.149	1.548

**Table 4:** Lattice mismatches among phases. * indicate that there are n cells in this particular crystallographic direction.

Commensurate Lattice Mismatches *m*	
δ-phase//γ-phase	m
6 *(0 2 2)//8 *(−1 3 1)	4.0%
(2 −1 1)//(1 −3 1)	1.0%
3 *(−1 −1 1)//4 *(3 1 0)	2.0%

## Data Availability

The datasets generated during and/or analyzed during the current study are available from the corresponding author on reasonable request.

## Supplementary Materials

The following are available online at https://www.mdpi.com/article/10.3390/nano11071823/s1: Figure S1: Gaussian Fit of the luminescence spectra collected on the samples a) CPI3-10m and b) CPI3-1h. Table S1: Fit parameters of PL spectrum in Figure S1a. Table S2: Fit parameters of PL spectrum in Figure S1b. Figure S2: Steady-state luminescence spectra of two different points on the samples a) CPI3-5h, b) CPI3-10h, c) CPI3-17h and d) CPI3-24h. Figure S3: PL Excitation spectra of the emission at 715 nm on the samples a) CPI3-1h, b) CPI3-5h and c) CPI3-10h.

## References

[1] Mitzi D.B. (2007). Synthesis, Structure, and Properties of Organic-Inorganic Perovskites and Related Materials. Progress in Inorganic Chemistry.

[2] Stranks S.D., Eperon G.E., Grancini G., Menelaou C., Alcocer M.J.P., Leijtens T., Herz L.M., Petrozza A., Snaith H.J. (2013). Electron-Hole Diffusion Lengths Exceeding 1 Micrometer in an Organometal Trihalide Perovskite Absorber. Science.

[3] Green M.A., Jiang Y., Soufiani A.M., Ho-Baillie A. (2015). Optical Properties of Photovoltaic Organic–Inorganic Lead Halide Perovskites. J. Phys. Chem. Lett..

[4] Xing G., Mathews N., Sun S., Lim S.S., Lam Y.M., Gratzel M., Mhaisalkar S., Sum T.C. (2013). Long-Range Balanced Electron- and Hole-Transport Lengths in Organic-Inorganic CH3NH3PbI3. Science.

[5] Walsh A. (2015). Principles of Chemical Bonding and Band Gap Engineering in Hybrid Organic–Inorganic Halide Perovskites. J. Phys. Chem. C.

[6] Kim H.-S., Lee C.-R., Im J.-H., Lee K.-B., Moehl T., Marchioro A., Moon S.-J., Humphry-Baker R., Yum J.-H., Moser J.E. (2012). Lead Iodide Perovskite Sensitized All-Solid-State Submicron Thin Film Mesoscopic Solar Cell with Efficiency Exceeding 9%. Sci. Rep..

[7] Cho H., Kim Y.-H., Wolf C., Lee H.-D., Lee T.-W. (2018). Improving the Stability of Metal Halide Perovskite Materials and Light-Emitting Diodes. Adv. Mater..

[8] Yang Z., Deng Y., Zhang X., Wang S., Chen H., Yang S., Khurgin J., Fang N.X., Zhang X., Ma R. (2018). High-Performance Single-Crystalline Perovskite Thin-Film Photodetector. Adv. Mater..

[9] Dong Q., Fang Y., Shao Y., Mulligan P., Qiu J., Cao L., Huang J. (2015). Electron-hole diffusion lengths >175 μm in solution-grown CH_3_ NH_3_ PbI_3_ single crystals. Science.

[10] Huang H., Polavarapu L., Sichert J.A., Susha A.S., Urban A.S., Rogach A.L. (2016). Colloidal lead halide perovskite nanocrystals: Synthesis, optical properties and applications. NPG Asia Mater..

[11] Anni M., Cretí A., De Giorgi M.L., De Lomascolo M. (2021). Local Morphology Effects on the Photoluminescence Properties of Thin CsPbBr3 Nanocrystal Films. Nanomaterials.

[12] Akbulatov A.F., Luchkin S.Y., Frolova L.A., Dremova N.N., Gerasimov K.L., Zhidkov I.S., Anokhin D.V., Kurmaev E.Z., Stevenson K.J., Troshin P.A. (2017). Probing the Intrinsic Thermal and Photochemical Stability of Hybrid and Inorganic Lead Halide Perovskites. J. Phys. Chem. Lett..

[13] Iagher L., Etgar L. (2018). Effect of Cs on the Stability and Photovoltaic Performance of 2D/3D Perovskite-Based Solar Cells. ACS Energy Lett..

[14] Zhou Y., Zhao Y. (2019). Chemical stability and instability of inorganic halide perovskites. Energy Environ. Sci..

[15] Deretzis I., Bongiorno C., Mannino G., Smecca E., Sanzaro S., Valastro S., Fisicaro G., Magna A.L., Alberti A. (2021). Exploring the structural competition between the black and the yellow phase of cspbi3. Nanomaterials.

[16] Stoumpos C.C., Malliakas C.D., Kanatzidis M.G. (2013). Semiconducting Tin and Lead Iodide Perovskites with Organic Cations: Phase Transitions, High Mobilities, and Near-Infrared Photoluminescent Properties. Inorg. Chem..

[17] Marronnier A., Roma G., Boyer-Richard S., Pedesseau L., Jancu J.M., Bonnassieux Y., Katan C., Stoumpos C.C., Kanatzidis M.G., Even J. (2018). Anharmonicity and Disorder in the Black Phases of Cesium Lead Iodide Used for Stable Inorganic Perovskite Solar Cells. ACS Nano.

[18] Mehdi H., Mhamdi A., Hannachi R., Bouazizi A. (2019). MAPbBr_3_ perovskite solar cells via a two-step deposition process. RSC Adv..

[19] Huang D., Xie P., Pan Z., Rao H., Zhong X. (2019). One-step solution deposition of CsPbBr_3_ based on precursor engineering for efficient all-inorganic perovskite solar cells. J. Mater. Chem. A.

[20] Konstantakou M., Perganti D., Falaras P., Stergiopoulos T. (2017). Anti-Solvent Crystallization Strategies for Highly Efficient Perovskite Solar Cells. Crystals.

[21] Wang P., Zhang X., Zhou Y., Jiang Q., Ye Q., Chu Z., Li X., Yang X., Yin Z., You J. (2018). Solvent-controlled growth of inorganic perovskite films in dry environment for efficient and stable solar cells. Nat. Commun..

[22] Li B., Zhang Y., Fu L., Yu T., Zhou S., Zhang L., Yin L. (2018). Surface passivation engineering strategy to fully-inorganic cubic CsPbI3 perovskites for high-performance solar cells. Nat. Commun..

[23] Luo P., Xia W., Zhou S., Sun L., Cheng J., Xu C., Lu Y. (2016). Solvent Engineering for Ambient-Air-Processed, Phase-Stable CsPbI_3_ in Perovskite Solar Cells. J. Phys. Chem. Lett..

[24] Xiang S., Fu Z., Li W., Wei Y., Liu J., Liu H., Zhu L., Zhang R., Chen H. (2018). Highly Air-Stable Carbon-Based α-CsPbI_3_ Perovskite Solar Cells with a Broadened Optical Spectrum. ACS Energy Lett..

[25] Protesescu L., Yakunin S., Bodnarchuk M.I., Krieg F., Caputo R., Hendon C.H., Yang R.X., Walsh A., Kovalenko M.V. (2015). Nanocrystals of Cesium Lead Halide Perovskites (CsPbX3, X = Cl, Br, and I): Novel Optoelectronic Materials Showing Bright Emission with Wide Color Gamut. Nano Lett..

[26] Ke W., Spanopoulos I., Stoumpos C.C., Kanatzidis M.G. (2018). Myths and reality of HPbI3 in halide perovskite solar cells. Nat. Commun..

[27] El Ajjouri Y., Palazon F., Sessolo M., Bolink H.J. (2018). Single-Source Vacuum Deposition of Mechanosynthesized Inorganic Halide Perovskites. Chem. Mater..

[28] Karmakar A., Dodd M.S., Zhang X., Oakley M.S., Klobukowski M., Michaelis V.K. (2019). Mechanochemical synthesis of 0D and 3D cesium lead mixed halide perovskites. Chem. Commun..

[29] Hong Z., Tan D., John R.A., Tay Y.K.E., Ho Y.K.T., Zhao X., Sum T.C., Mathews N., García F., Soo H. (2019). Sen Completely Solvent-free Protocols to Access Phase-Pure, Metastable Metal Halide Perovskites and Functional Photodetectors from the Precursor Salts. iScience.

[30] Straus D.B., Guo S., Cava R.J. (2019). Kinetically Stable Single Crystals of Perovskite-Phase CsPbI_3_. J. Am. Chem. Soc..

[31] Satta J., Melis C., Carbonaro C.M., Pinna A., Salado M., Salazar D., Ricci P.C. (2021). Raman spectra and vibrational analysis of CsPbI3: A fast and reliable technique to identify lead halide perovskite polymorphs. J. Mater..

[32] Lutterotti L. (2010). Total pattern fitting for the combined size–strain–stress–texture determination in thin film diffraction. Nucl. Instrum. Methods Phys. Res. Sect. B Beam Interact. Mater. Atoms.

[33] Zhang Q., Zhou Y., Wei Y., Tai M., Nan H., Gu Y., Han J., Yin X., Li J., Lin H. (2020). Improved phase stability of γ-CsPbI _3_ perovskite nanocrystals using the interface effect using iodine modified graphene oxide. J. Mater. Chem. C.

[34] Sutton R.J., Filip M.R., Haghighirad A.A., Sakai N., Wenger B., Giustino F., Snaith H.J. (2018). Cubic or Orthorhombic? Revealing the Crystal Structure of Metastable Black-Phase CsPbI_3_ by Theory and Experiment. ACS Energy Lett..

[35] Yang Y., Robbins J.P., Ezeonu L., Ma Y., Sparta N., Kong X., Strauf S., Podkolzin S.G., Lee S.S. (2020). Probing lattice vibrations of stabilized CsPbI_3_ polymorphs via low-frequency Raman spectroscopy. J. Mater. Chem. C.

[36] Liu F., Zhang Y., Ding C., Kobayashi S., Izuishi T., Nakazawa N., Toyoda T., Ohta T., Hayase S., Minemoto T. (2017). Highly Luminescent Phase-Stable CsPbI _3_ Perovskite Quantum Dots Achieving Near 100% Absolute Photoluminescence Quantum Yield. ACS Nano.

[37] Zhao B., Jin S.-F., Huang S., Liu N., Ma J.-Y., Xue D.-J., Han Q., Ding J., Ge Q.-Q., Feng Y. (2018). Thermodynamically Stable Orthorhombic γ-CsPbI_3_ Thin Films for High-Performance Photovoltaics. J. Am. Chem. Soc..

[38] Babin V., Krasnikov A., Nikl M., Nitsch K., Stolovits A., Zazubovich S. (2003). Luminescence and relaxed excited state origin in CsI:Pb crystals. J. Lumin..

[39] Chen C., Zhang L., Shi T., Liao G., Tang Z. (2019). Controllable synthesis of all inorganic lead halide perovskite nanocrystals with various appearances in multiligand reaction system. Nanomaterials.

[40] Lakowicz J.R., Lakowicz J.R. (2006). Principles of Fluorescence Spectroscopy.

[41] Becker P., Márquez J.A., Just J., Al-Ashouri A., Hages C., Hempel H., Jošt M., Albrecht S., Frahm R., Unold T. (2019). Low Temperature Synthesis of Stable γ-CsPbI_3_ Perovskite Layers for Solar Cells Obtained by High Throughput Experimentation. Adv. Energy Mater..

