# Supplementary material for: Formation Mechanisms and Phase Stability of Solid-State Grown CsPbI3 Perovskites
Source: Nanomaterials (Basel). 2021 Jul 14;11(7):1823. doi: 10.3390/nano11071823 (PMC8308418; doi:10.3390/nano11071823)
Supplement: Supplementary file 1 [file nanomaterials-11-01823-s001.zip › nanomaterials-1275938-SI.pdf]

Supplementary Materials

Formation Mechanisms and Phase Stability of Solid-State Grown $CsPbI_3$ Perovskites

Jessica Satta [1], Alberto Casu [2], Daniele Chiriu [1], Carlo Maria Carbonaro [1], Luigi Stagi [3] and Pier Carlo Ricci [1,*]

[1] Department of Physics, University of Cagliari, Campus of Monserrato, 09042 Monserrato, Italy; jessica.satta@dsf.unica.it (J.S.); daniele.chiriu@dsf.unica.it (D.C.); cm.carbonaro@dsf.unica.it (C.M.G.)
[2] Biological and Environmental Sciences and Engineering (BESE) Division, King Abdullah University of Science and Technology (KAUST), Nabla Lab, Thuwal 23955-6900, Saudi Arabia; alberto.casu@kaust.edu.sa
[3] Laboratory of Materials Science and Nanotechnology, CR-INSTM, Department of Chemistry and Pharmacy, University of Sassari, Via Vienna 2, 07100 Sassari, Italy; lstagi@uniss.it
* Correspondence: carlo.ricci@dsf.unica.it

Gaussian fit of luminescence peaks

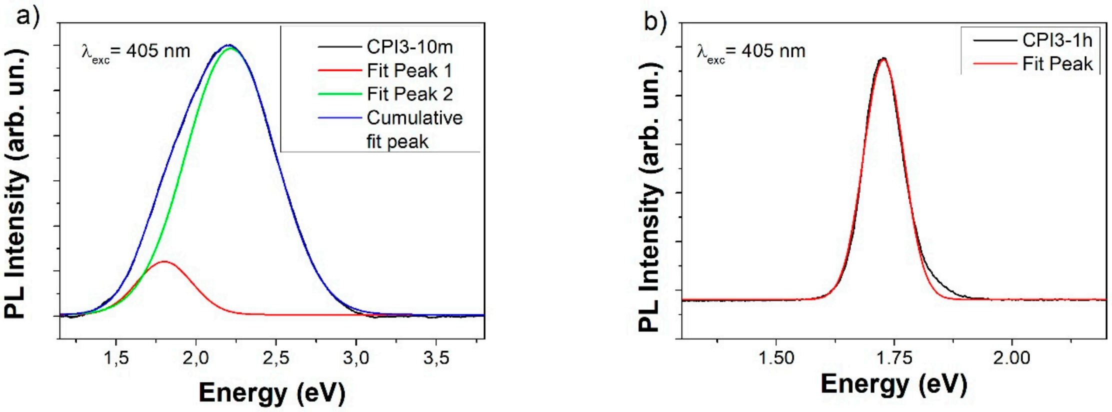

Figure S1. Gaussian Fit of the luminescence spectra collected on the samples a) CPI3-10m and b) CPI3-1h.

Figure S1 reports the gaussian fit of the two luminescence peaks. The broad emission of the d-$CsPbI_3$ is the convolution of two gaussian peaks:

$$I = I_0 + A_1 e^{-\frac{(x-x_{c1})^2}{2w_1^2}} + A_2 e^{-\frac{(x-x_{c2})^2}{2w_2^2}}$$

with A amplitude, x_c the centre and w the width of the peak.
Retrieved parameters are reported in Table S1.

Table S1. Fit parameters of PL spectrum in figure S1a.

	A_i	x_c (eV)	w (eV)	FWHM (eV)
**Peak 1**	59 ± 3	1.802 ± 0.004	0.172 ± 0.003	0.406 ±0.008
**Peak 2**	294.5 ± 0.9	2.216 ± 0.003	0.280 ± 0.002	0.659 ±0.004

The narrow emission around 715 nm can be fitted with a single gaussian peak:

$$I = I_0 + A e^{-\frac{(x-x_c)^2}{2w^2}},$$

Retrieved parameters are reported in Table S2.

Table S2. Fit parameters of PL spectrum in figure S1b.

	A_i	x_c (eV)	w (eV)	FWHM (eV)
**Peak 1**	4939 ± 9	1.72734 ± 0.00009	0.04240 ± 0.00009	0.0998 ±0.0002

Steady state luminescence

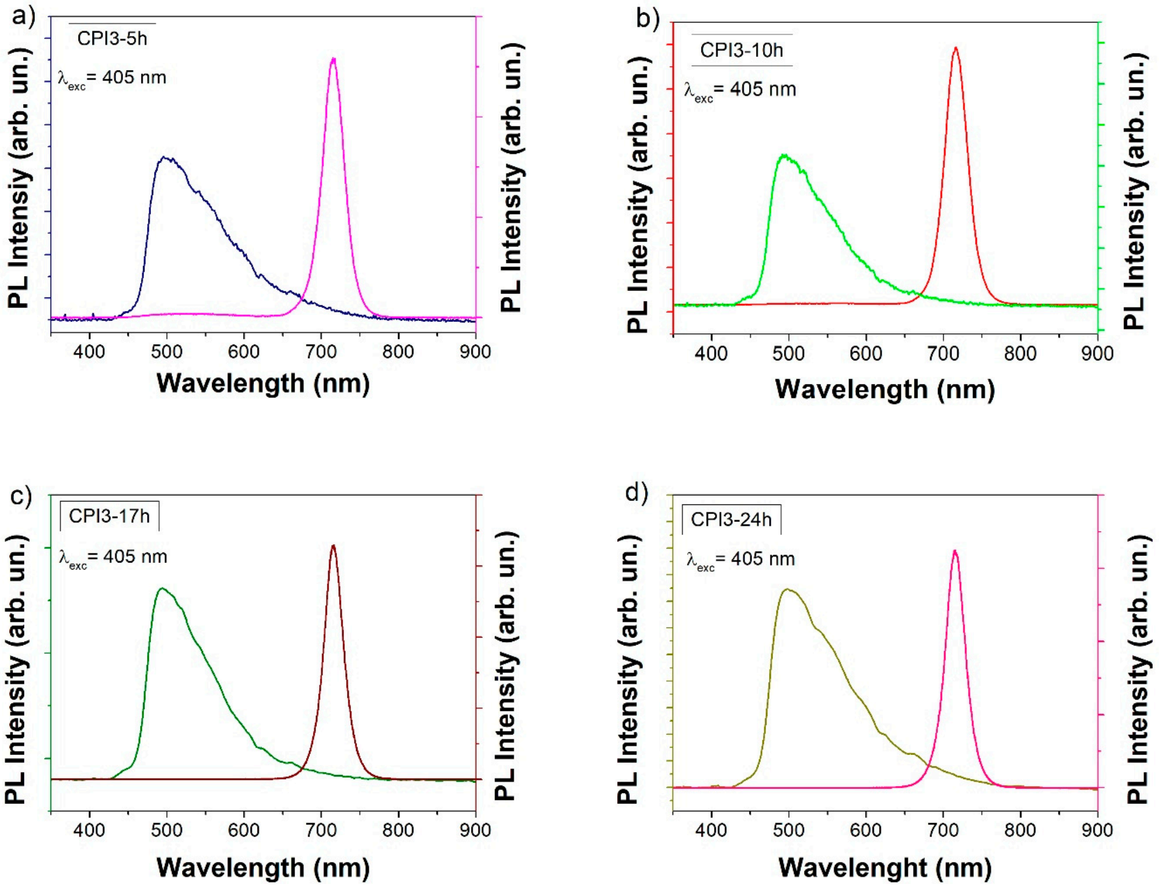

Figure S2. Steady-state luminescence spectra of two different points on the samples a) CPI3-5h, b) CPI3-10h, c) CPI3-17h and d) CPI3-24h.

Figure S2 shows steady-state luminescence spectra collected on different points of the samples CPI3-5h, CPI3-10h, CPI3-17h and CPI3-24h. The graphs confirm the inhomogeneity of the samples, showing that the luminescence is point-dependent. It's possible to observe a broad peak around 550 nm and a narrow one around 715 nm.

Excitation spectra for the peak at 715 nm

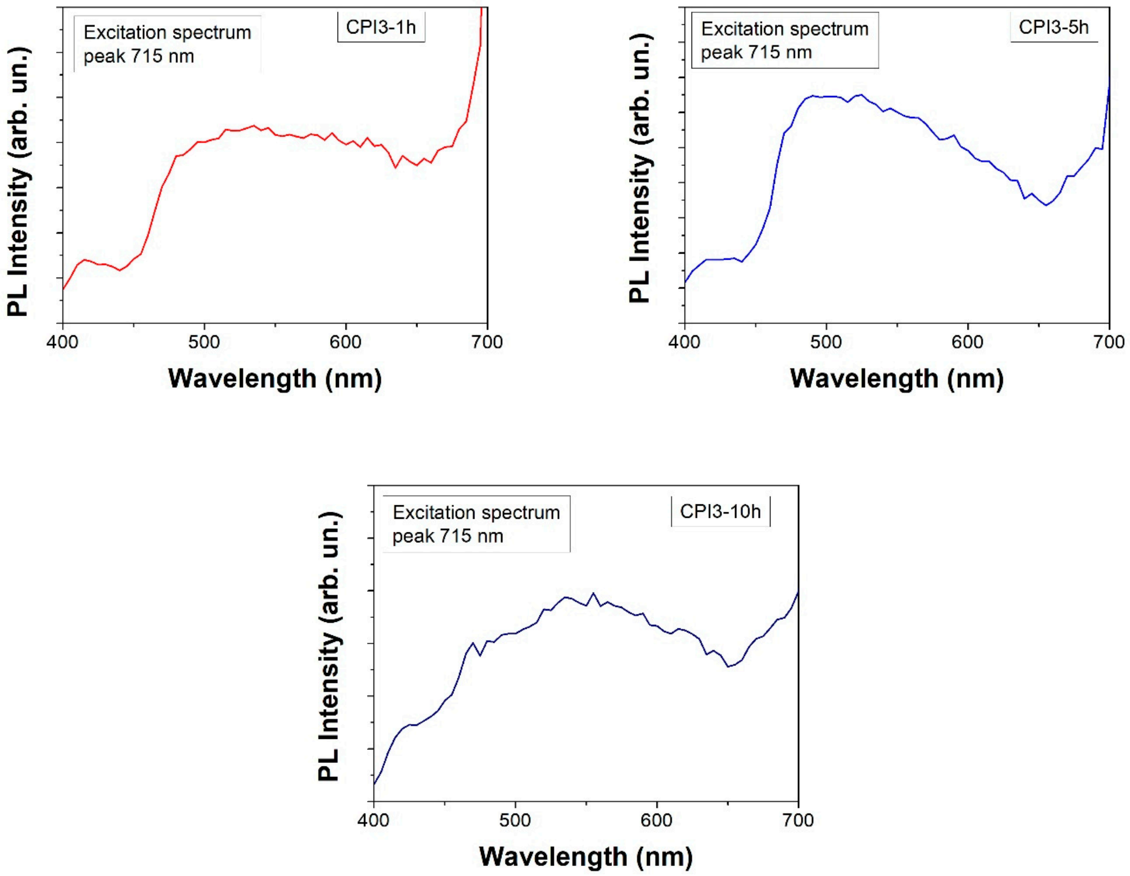

Figure S3. PL Excitation spectra of the emission at 715 nm on the samples a) CPI3-1h, b) CPI3-5h and c) CPI3-10h.

Figure S3 shows the PL Excitation spectra of the emission at 715 nm of the samples CPI3-1h, CPI3-5h and CPI3-10h. The spectra have been extrapolated from the 3D maps in Figure 8.
